# 3D whole-heart grey-blood late gadolinium enhancement cardiovascular magnetic resonance imaging

**DOI:** 10.1186/s12968-021-00751-2

**Published:** 2021-05-24

**Authors:** Giorgia Milotta, Camila Munoz, Karl P. Kunze, Radhouene Neji, Stefano Figliozzi, Amedeo Chiribiri, Reza Hajhosseiny, Pier Giorgio Masci, Claudia Prieto, René M. Botnar

**Affiliations:** 1 School of Biomedical Engineering and Imaging Sciences, King’s College London, St Thomas’ Hospital (3rd Floor - Lambeth Wing), Westminster Bridge Road, London, SE1 7EH UK; 2 MR Research Collaborations, Siemens Healthcare Limited, Frimley, UK; 3 Escuela de Ingeniería, Pontificia Universidad Católica de Chile, Santiago, Chile

**Keywords:** 3D whole-heart, Respiratory motion correction, Late gadolinium enhancement, Dixon water/fat separation

## Abstract

**Purpose:**

To develop a free-breathing whole-heart isotropic-resolution 3D late gadolinium enhancement (LGE) sequence with Dixon-encoding, which provides co-registered 3D grey-blood phase-sensitive inversion-recovery (PSIR) and complementary 3D fat volumes in a single scan of < 7 min.

**Methods:**

A free-breathing 3D PSIR LGE sequence with dual-echo Dixon readout with a variable density Cartesian trajectory with acceleration factor of 3 is proposed. Image navigators are acquired to correct both inversion recovery (IR)-prepared and reference volumes for 2D translational respiratory motion, enabling motion compensated PSIR reconstruction with 100% respiratory scan efficiency. An intermediate PSIR reconstruction is performed between the in-phase echoes to estimate the signal polarity which is subsequently applied to the IR-prepared water volume to generate a water grey-blood PSIR image. The IR-prepared water volume is obtained using a water/fat separation algorithm from the corresponding dual-echo readout. The complementary fat-volume is obtained after water/fat separation of the reference volume. Ten patients (6 with myocardial scar) were scanned with the proposed water/fat grey-blood 3D PSIR LGE sequence at 1.5 T and compared to breath-held grey-blood 2D LGE sequence in terms of contrast ratio (CR), contrast-to-noise ratio (CNR), scar depiction, scar transmurality, scar mass and image quality.

**Results:**

Comparable CRs (*p* = 0.98, 0.40 and 0.83) and CNRs (*p* = 0.29, 0.40 and 0.26) for blood-myocardium, scar-myocardium and scar-blood respectively were obtained with the proposed free-breathing 3D water/fat LGE and 2D clinical LGE scan. Excellent agreement for scar detection, scar transmurality, scar mass (bias = 0.29%) and image quality scores (from 1: non-diagnostic to 4: excellent) of 3.8 ± 0.42 and 3.6 ± 0.69 (*p* > 0.99) were obtained with the 2D and 3D PSIR LGE approaches with comparable total acquisition time (*p* = 0.29). Similar agreement in intra and inter-observer variability were obtained for the 2D and 3D acquisition respectively.

**Conclusion:**

The proposed approach enabled the acquisition of free-breathing motion-compensated isotropic-resolution 3D grey-blood PSIR LGE and fat volumes. The proposed approach showed good agreement with conventional 2D LGE in terms of CR, scar depiction and scan time, while enabling free-breathing acquisition, whole-heart coverage, reformatting in arbitrary views and visualization of both water and fat information.

**Supplementary Information:**

The online version contains supplementary material available at 10.1186/s12968-021-00751-2.

## Introduction

Late gadolinium enhancement (LGE) cardiovascular magnetic resonance (CMR) plays an important role in the assessment of ischemic heart diseases and myocardial viability [1, 2]. Accurate delineation of LGE enables to gauge scar transmurality and burden in patients with chronic ischemic cardiomyopathy [3, 4], which plays a key role in guiding coronary artery revascularization. Conventionally, LGE imaging relies on T1-weighted 2D images acquired in different orientations (i.e. short-axis, 2-chamber, 3-chamber and 4-chamber views) 10–20 min after the intravenous injection of a gadolinium-based contrast agent (GBCA). The acquisition scheme usually consists of an inversion recovery (IR) pulse followed by a waiting time, called inversion time (TI), and signal readout. With this approach the TI is usually chosen to null the signal from healthy myocardium, enhancing the infarcted myocardium due to the retention of GBCA [5, 6]. This so-called “bright-blood” method achieves high contrast between ischemic and healthy myocardium, however the bright signal from the adjacent blood pool hinders scar delineation at the blood-scar border [7]. Additionally, heart rate variations during the acquisition can impair the choice of the TI, resulting in suboptimal scar visualization [8].

Phase-sensitive inversion-recovery (PSIR) sequences mitigate the effects of suboptimal selection of TI [9]. PSIR sequences acquire an IR-prepared image every other heartbeat, while during the second heartbeat a reference image is acquired with a low flip angle. This reference image is used for coil sensitivity normalization and in the PSIR reconstruction to determine the phase of the signal acquired during the first (IR-prepared) heartbeat. The signal polarity of the longitudinal magnetization is taken into account during the reconstruction of the PSIR image. Therefore, signal with negative longitudinal magnetization appears black in the PSIR image, signal with positive longitudinal magnetization appears bright in the image, whereas nulled signal appears grey [9, 10]. In contrast, in conventional magnitude reconstruction signals with both negative and positive longitudinal magnetization appear bright in the image and the nulled signal appears black. PSIR reconstruction extends the greyscale range for visualization avoiding the requirement of precise nulling. Healthy myocardium signal is commonly nulled in PSIR acquisitions, resulting in a bright-blood PSIR LGE image that still can suffer from suboptimal contrast between blood and scar [11].

Several approaches, with and without PSIR reconstruction, have been proposed to increase the scar-to-blood contrast by simultaneously nulling healthy myocardium and blood signals. These so called “dark-blood” methods are based on the combination of several preparation pulses such as double IR pulses [12–14], T2 preparation (T2prep) and IR [7, 15–17], or magnetization transfer and IR [18, 19]. Although improved scar visualization is obtained with these approaches, the addition of magnetization preparation pulses leads to a more sophisticated sequence planning and requires a dedicated Look-Locker scout sequence matching the magnetization preparation scheme.

Recently, a grey-blood 2D PSIR LGE sequence that does not use additional preparation pulses has shown promising and robust results in scar depiction due to increased contrast between scar, normal myocardium and blood [11, 20]. This sequence optimized the TI to null blood signal instead of healthy myocardium, leading to an improved scar-to-blood contrast with respect to bright-blood LGE sequences, but maintaining high scar-to-myocardium contrast. Similarly to conventional bright-blood PSIR imaging, 2D grey-blood PSIR LGE images are acquired over several breath-holds in different image orientations to visualise and quantify scar extension. However, this approach is limited by potential image misalignment between different slices, anisotropic resolution and the need for multiple breath-holds.

Recently free-breathing 3D LGE imaging techniques have been proposed [21–25] to overcome the limitations related to 2D breath-hold LGE. These techniques used magnitude-based reconstruction, thus image quality may be affected by suboptimal TI selection. 3D PSIR LGE has been proposed to overcome this limitation, however these approaches suffer from unpredictable scan times associated with the use of diaphragmatic respiratory navigation [22, 23]. Additionally, fat-suppression in 3D LGE may be challenging due to field inhomogeneities, thus fat induced artefacts and presence of myocardial fat infiltration or epicardial fat may render the distinction between fat and LGE challenging both in ischemic and non-ischemic cardiomyopathies [10, 26]. Water/fat LGE approaches have been proposed to enable visualization of both scar and fat tissues, however these techniques suffer from limited spatial resolution [26–30].

In this work we propose a novel free-breathing motion corrected whole-heart 3D PSIR LGE prototype sequence with water/fat Dixon encoding and magnitude blood-nulling which provides a grey-blood PSIR volume for scar visualization, and complementary fat detection on 3D fat images. The proposed approach is based on the acquisition of two interleaved gradient echo (GRE) datasets with Dixon readout. The first dataset is acquired with an IR pulse with optimized TI to null the blood signal, whereas the second dataset is acquired with no preparation and a low flip angle, and is used as reference volume for the PSIR reconstruction. Image navigators are acquired to correct both IR-prepared and reference volumes for 2D translational respiratory motion, enabling motion compensated grey-blood PSIR reconstruction with 100% respiratory scan efficiency. The 3D isotropic nature of the acquisition enables reformatting the acquired volumes in any desired imaging plane.

## Methods

### 3D grey-blood PSIR LGE framework

**Fig. 1:**
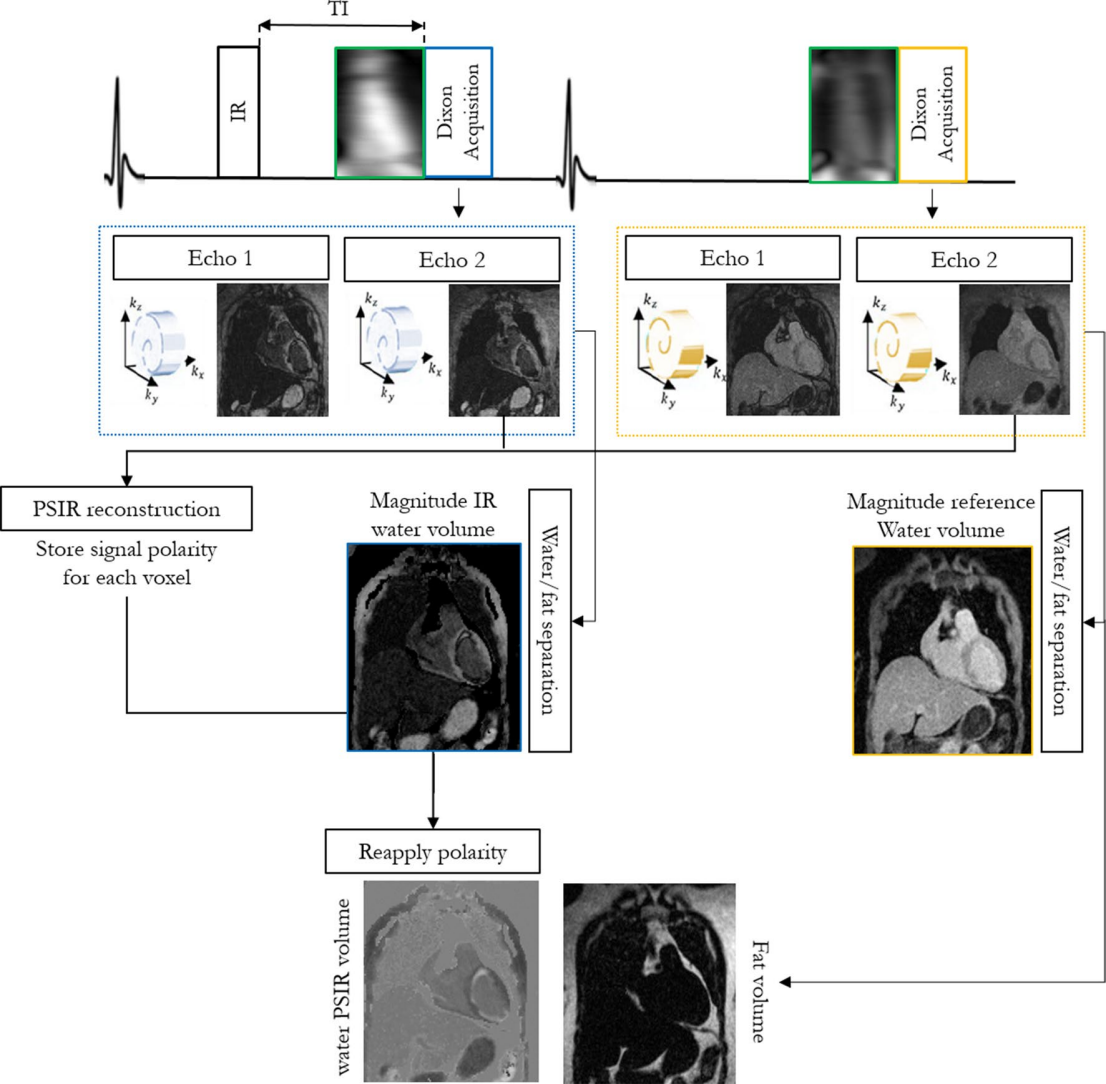
Framework of the proposed 3D grey-blood phase sensitive inversion recovery (PSIR) late gadolinium enhancement (LGE) sequence. Two interleaved gradient echo (GRE) volumes with two-point Dixon encoding and 3 × undersampled spiral-like variable density Cartesian trajectory (VD-CASPR) are acquired with IR-preparation and no preparation respectively. Image navigators (iNAVs) are acquired prior to the 3D acquisition to correct for translational respiratory motion. The four echoes are motion corrected to end-expiration and reconstructed with iterative-SENSE. A water/fat separation algorithm is then used to obtain water and fat images for each acquired volume. An intermediate PSIR reconstruction between the in-phase echoes of each acquired volume is performed to estimate the signal polarity which is subsequently applied to the inversion recovery (IR)-prepared water volume to generate a water grey-blood PSIR image. The complementary fat-volume is obtained after water/fat separation of the reference volume

The framework of the proposed free-breathing grey-blood whole-heart 3D PSIR LGE prototype sequence is shown in Fig. 1. Two interleaved 3D volumes are acquired with electrocardiogram (ECG)-triggered spoiled GRE readout and 3 × undersampled variable-density Cartesian spiral-like trajectory [31, 32]. The first volume is acquired with an IR pulse with patient dependent TI set to null the blood signal, whereas the second dataset is acquired with no preparation pulses. A bipolar GRE Dixon readout is used to achieve water/fat separation in the two interleaved datasets irrespective of the magnetization preparation and to increase the signal-to-noise ratio (SNR) of the water images.

2D low-resolution coronal image navigators (iNAVs) were acquired prior to the acquisition of each dual-echo volume to track and correct for the translational superior-inferior (SI) and left–right (LR) heart motion induced by respiration, enabling 100% respiratory scan efficiency and predictable scan time (no data rejection). For each volume the corresponding out-of-phase iNAVs were used to estimate the respiratory motion displacements, using a template matching algorithm [33], thus avoiding contrast variation between the iNAVs acquired with different magnetization preparation [34]. Motion correction to end expiration (linear phase-shift in k-space) [35] was performed for the dual-echo (in and out-of-phase) IR prepared and reference volumes independently. The four 3D motion compensated undersampled in and out-of-phase datasets were reconstructed with iterative SENSE [36], and a rigid image registration between the four motion corrected echoes was performed.

A water/fat separation algorithm with magnitude based B_0_ estimation and phase unwrapping (B0-NICEbd) [37] was used to generate the water and fat volumes for each acquired set. After water/fat separation the signal polarity is lost and thus is not possible to obtain the grey-blood PSIR volume directly from the resulting water images. An intermediate PSIR [9] reconstruction is performed between the two in-phase datasets prior to water/fat separation to estimate the signal polarity which is subsequently reapplied to the IR-prepared magnitude water volume to generate a water grey-blood PSIR image. This allowed to obtain a water PSIR volume in which blood and fat signal appear grey due to their nulling in the magnitude IR-prepared water image, healthy myocardium appears dark and scar appears bright. The complementary volume for fat visualization is obtained from the reference dataset (no magnetization preparation) because of the high signal-to-noise.

### Experiments

The proposed free-breathing grey-blood 3D LGE prototype sequence was tested in a T1 phantom and 10 patients (mean 65 years, range: 54*–*79 years; 6 male) with known or suspected cardiovascular disease and compared against conventional breath-hold grey-blood 2D LGE imaging with magnitude blood-nulling and PSIR reconstruction. Acquisitions were performed on a 1.5 T CMR scanner (MAGNETOM Aera, Siemens Healthineers, Erlangen, Germany) with an 18-channel chest coil and a 32-channel spine coil. Written informed consent was obtained from all participants before CMR and the study was approved by the Institutional Review Board.

#### Phantom

Data acquisition was performed with the proposed 3D PSIR LGE prototype sequence using an in-house developed T1 phantom composed of 7 vials filled with different gadolinium concentrations. The phantom experiment was performed to evaluate the reliability of the Look-Locker scout scan for TI selection and to compare the contrast-ratio (CR) and contrast-to-noise ratio (CNR) between myocardium-blood, scar-myocardium and scar-blood on 3D PSIR LGE images obtained for both magnitude blood-nulling and conventional magnitude myocardium-nulling. Comparison between the 3D magnitude blood-nulling (grey-blood PSIR LGE) and 3D myocardial-nulling (bright-blood PSIR LGE) was carried out to evaluate the capability of the proposed 3D framework to replicate the results in terms of CR and CNR obtained in [20]. The T1 phantom included vials with gadolinium concentrations of 0, 0.1, 0.3, 0.5, 0.7, 0.9, and 1 mMol. A reference 2D Inversion-Recovery Spin Echo (IRSE) experiment was performed to quantify the T1 values of the phantom vials (Table 1). Acquisition parameters for the IRSE sequence included transversal orientation, FOV = 180 × 180 × 16 mm^3^, resolution = 2 × 2 × 4 mm^3^, TR = 10 s, TE = 1 ms, TIs = [50, 100, 150, 300, 500, 1000, 2000, 3000]ms and total scan time TA = 4 h 32 min. The vials with gadolinium concentration of 0.3mMol, 0.5mMol and 0.7mMol showed T1 values corresponding approximately to post-contrast myocardium, blood and scar respectively (T1s of 587 ms, 384 ms and 279 ms) with respect to post-contrast T1 values reported in a standardized T1 phantom (T1_blood_ = 458 ms, T1_myoc_ = 430 ms and T1_scar_ = 300 ms) [38] and thus were considered for the CR and CNR analysis.

**Table 1:** Gadolinium concentration, T1 values and inversion time (TI) used to null the signal in the IR-prepared image for each phantom vial

Gd [mMol]	T1 [ms]	TI [ms]
0.0 (H_2_0)	2883	–
0.1	1259	630
0.3	587	395
0.5	384	240
0.7	279	145
0.9	217	130
1	198	130

TI optimization was not performed to null the vial with 0.0 mMol of gadolinium concentration

The proposed 3D PSIR LGE acquisition parameters included transversal orientation, GRE readout with two-point Dixon bipolar encoding and centric k-space reordering, FA = 25 deg and 5 deg for IR-prepared and non-prepared dataset respectively, isotropic resolution of 2 mm^3^, FOV = 200 × 200 × 20 mm^3^, 14 low flip angle echoes (FA = 3 deg) for iNAV acquisition, TR/TE1/TE2 = 6.41/2.38/4.76 ms, bandwidth = 602 Hz/pixel, 16 segments per heart beat corresponding to an acquisition window of 107 ms, simulated heart rate of 60 bpm, and acceleration factor of 3 for each acquired volume leading to a total acquisition time of ~ 2 min. A 2D TI Look-Locker scout scan with imaging parameters matching the 3D PSIR LGE sequence was performed prior to acquisition to select the optimal TI to null the signal from phantom vials including those corresponding to blood and myocardium.

#### Patients

Data were acquired with the proposed free-breathing grey-blood 3D PSIR LGE prototype sequence in 10 patients who underwent CMR due to suspected cardiovascular disease. The proposed ECG-triggered 3D sequence was acquired during mid-diastole, in coronal orientation with dual-echo Dixon bipolar GRE readout, isotropic resolution of 2mm^3^, FOV = 320 × 320 × 96-140 mm^3^, FA = 25 deg for the IR-prepared dataset and 5 deg for the reference dataset, 14 echoes with FA = 3 deg were used for iNAV acquisitions, TR/TE1/TE2 = 6.41/2.38/4.76 ms, bandwidth = 602 Hz/pixel, and acceleration factor of 3 for each volume.

The proposed 3D PSIR LGE sequence was compared with the conventional breath-hold grey-blood 2D PSIR LGE sequence acquired during the clinical scan at our institution. The 2D LGE acquisition parameters included: balanced steady state free precession (bSSFP) readout with FA = 45 deg, in-plane resolution = 1.4 × 1.4 mm^2^, slice thickness = 8 mm, and 12 s breath-hold per slice. Images were acquired in 2-chamber, 3-chamber and 4-chamber orientations, and short-axis orientation (13–15 slices), requiring overall 16–18 breath-holds.

Grey-blood 2D and 3D PSIR LGE sequences were acquired respectively 9 min 24 s (range 8–13 min) and 25 min 42 sec (range 18–29 min) after a 0.15 mml/Kg bolusmmol/kg of GbCA (Gadobutrol, Gadovist, Bayer Healthcare, Berlin, Germany). 2D TI Look-Locker scout scans with imaging parameters matching respectively the 2D and the 3D LGE sequences were performed in each patient prior LGE imaging to select the optimal TI to null left ventricular (LV) blood signal for both 2D and 3D LGE sequences. The mean optimal TI for the 2D sequence was 159 ms with a range of 135 to 190 ms, whereas the mean optimal TI for the 3D acquisition was 197 ms with a range of 140 to 230 ms.

Data acquisition was performed during mid-diastole in all patient acquisitions. A breath-held 4-chamber CINE scan was performed at the beginning of the acquisition prior to the LGE protocol to select the patient specific trigger delay that corresponded with the diastolic quiescent period. Trigger delay ranged between 462 and 832 ms, whereas acquisition window ranged between 102 and 115 ms corresponding to 16 and 18 segments, respectively.

### Reconstruction

The 2D LGE magnitude images and grey-blood 2D LGE PSIR images were reconstructed directly on the scanner with inline software (Syngo MR VE11C, Siemens Healthineers) as described in [9].

Grey-blood 3D PSIR LGE and fat volumes were reconstructed offline using MATLAB R2017a (The MathWorks, Inc., Natick, Massachusetts, USA) on a dedicated workstation (16-core Dual Intel Xeon Processor, 2.3 GHz, 256 GB RAM). The total reconstruction time averaged 7 min 10 s.

### Data analysis

#### Phantom

The reliability of the 2D TI Look-Locker scout scan was tested by optimizing different TIs to null each vial of the phantom, excluding the vial with 0 mMol gadolinium concentration. CR and CNR between blood-myocardium, scar-myocardium and scar-blood was measured in the 3D PSIR LGE reconstructed images obtained with magnitude myocardium-nulling (TI = 395 ms) and the proposed magnitude blood-nulling approach (TI = 240 ms).

#### Patients

The proposed free-breathing grey-blood 3D PSIR LGE sequence was compared to conventional breath-hold grey-blood 2D PSIR LGE in terms of CR and CNR between blood-myocardium, scar-myocardium and scar-blood, scar detection, scar transmurality assessment, scar mass quantification, image quality and total acquisition time. The grey-blood 3D LGE volumes were manually reformatted in short axis, matching the 2D acquisition orientation, using OsiriX software (version MD 9.5, Osirix Foundation, Geneva, Switzerland)) with linear interpolation. Scar detection, scar transmurality assessment, scar mass quantification and image quality assessment were performed by two expert cardiologists (cardiologist 1 (PGM): 14 years of experience in CMR, SCMR level 3, cardiologist 2 (RH): 2 years of experience in CMR, SCMR level 2) for both grey-blood 2D and 3D PSIR LGE short-axis stacks in randomized order and with no side by side comparison between 2 and 3D images on per patient basis. 2D short-axis stack included 13–15 slices per patient, whereas the 3D short-axis volume included a mean of 59 slices (range: 42*–*76) covering the entire LV. All slices covering the LV were analysed by both observers for both the 2D and 3D acquisitions. Scar detection and scar transmurality analysis were carried out using the 17-segment American Heart Association (AHA) model [39]. The 17 LV segments were visually identified independently on the 2D and 3D short-axis images by each observer, and scar detection and scar transmurality assessment were visually performed by each cardiologist independently for each segment. Five transmurality scores (0%, 1–25%, 26–50%, 51–75%, 76–100%) were assigned to each segment of the 17 segment AHA model for both the 2D and 3D grey-blood LGE PSIR datasets.

Scar mass quantification was performed independently by each cardiologist by manually segmenting the scar on both 2D and 3D volumes for each scarred patient with cvi42 software (Circle Cardiovascular Imaging, Calgary, Alberta, Canada). The scar mass was expressed as percentage of total left ventricular (LV) mass, and the comparison between 2 and 3D measurement was performed with a Bland Altman analysis.

Image quality score assessment was performed using a 4-point Likert scale [40] including: 1: non-diagnostic quality, 2: poor diagnostic quality, 3: good diagnostic quality and 4: excellent diagnostic quality. A Student’s *t*-test was used to compare differences in continuous variables (CR, CNR, scar mass and total acquisition time) between the two groups (2D and 3D LGE PSIR). Scar mass differences between 2 and 3D LGE PSIR acquisition were additionally compared in a Bland Altman analysis. Scar detection and scar transmurality variations between 2 and 3D LGE PSIR images were assessed using a Cohen’s kappa test. Image quality scores were compared with a paired Wilcoxon signed-rank test to assess statistical differences. Statistical tests were two-tailed with p < 0.05 considered statistically significant. Intra-observer variability was assessed for scar detection and image quality measurements, whereas inter-observer variability was assessed for scar detection, scar transmurality, scar mass and image quality. Cohen’s kappa test was used to assess both intra and inter-observer variabilities, whereas Bland Altman analysis was used to assess inter-observer variability of scar mass quantification.

## Results

### Phantom

**Fig. 2:**
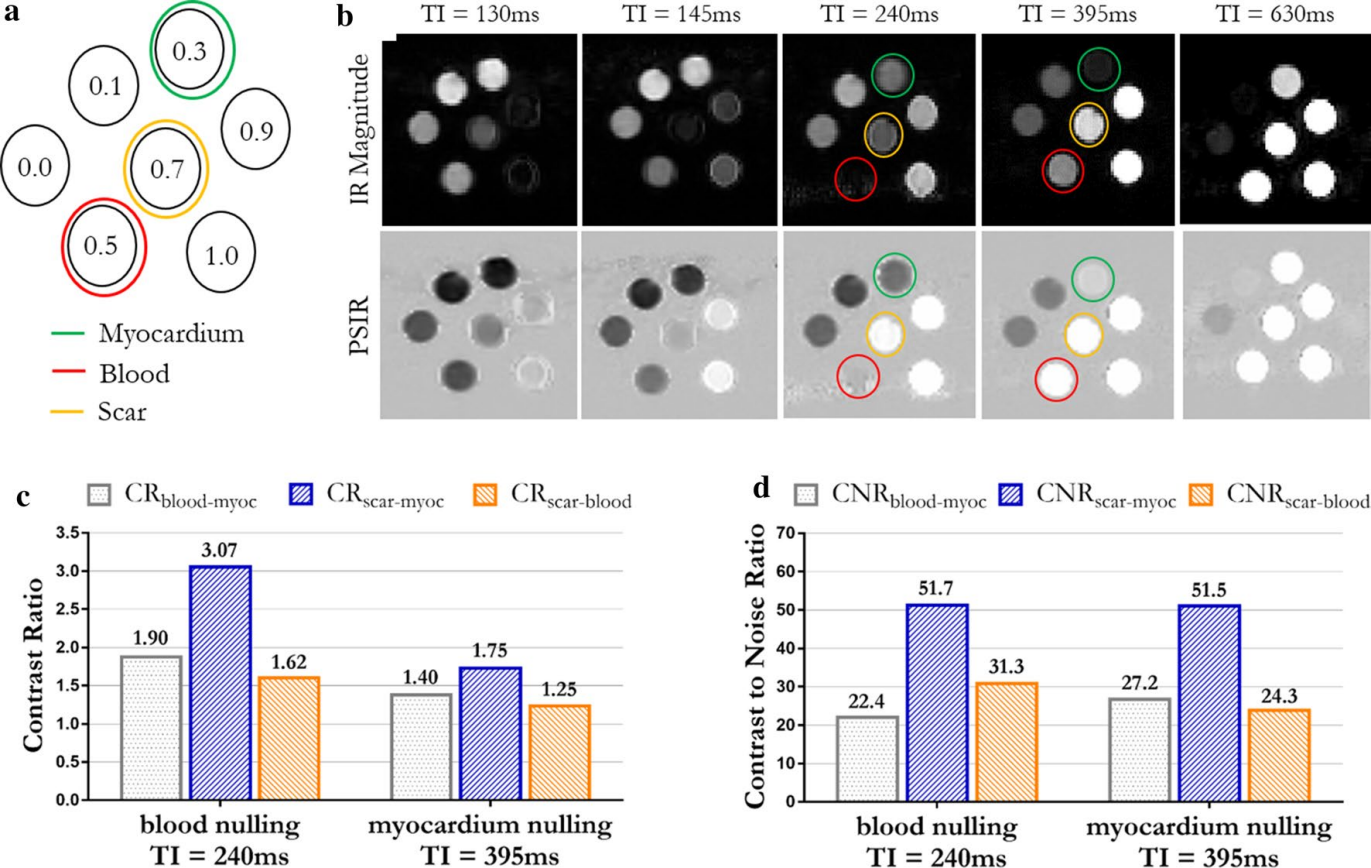
**a** Gadolinium concentrations and disposition of the phantom vials. Concentrations of 0.3, 0.5 and 0.7 mMol corresponded respectively to the post-contrast T1 of healthy myocardium, blood and scar. **b** 3D IR magnitude images and PSIR reconstruction obtained by nulling with different inversion times each phantom vial (excluding the one with 0 mMol concentration). TI = 240 ms corresponds to blood nulling, TI = 395 ms corresponds to myocardium nulling. **c** Contrast ratio (CR) between myocardium, blood and scar obtained with blood and myocardium nulling. **d** Contrast to noise ratio (CNR) between myocardium, blood and scar obtained with blood and myocardium nulling

Good nulling of the different phantom vials was obtained by optimizing the TI with the 2D Look-Locker TI scout scan. The optimized TI ranged between 130 and 630 ms for vials with GBCA concentration of 1 mMol and 0.1 mMol respectively. The optimal TIs for the nulling of each phantom vial are shown in Table 1, whereas the magnitude and PSIR images reconstructed for each 3D LGE acquisition are shown in Fig. 2b.

CR and CNR analysis were carried out on the PSIR images reconstructed for magnitude blood-nulling (TI = 240 ms) and magnitude myocardium-nulling (TI = 395 ms) (Fig. 2c, d). Higher CRs between blood-myocardium, scar-myocardium and scar-blood were observed with the proposed blood-nulling PSIR LGE with respect to the myocardial-nulling approach. Particularly CR_blood-myoc_ = 1.90, CR_scar-myoc_ = 3.07 and CR_scar-blood_ = 1.62 were measured in the grey-blood PSIR image, whereas CR_blood-myoc_ = 1.40, CR_scar-myoc_ = 1.75 and CR_scar-blood_ = 1.25 were measured in the bright-blood PSIR acquisition (Fig. 2c). Similar CNR between vials corresponding to myocardium and scar was observed with blood-nulling and myocardial-nulling approaches (CR_scar-myoc_ = 51.7 and 51.5 respectively). A slightly lower CNR between vials corresponding to myocardium and blood was observed with the myocardium-nulling approach (CNR_myoc-blood_ = 22.4) in comparison to blood-nulling (CNR_myoc-blood_ = 27.2), whereas an increased scar-to-blood CNR was observed with blood-nulling in vials corresponding to scar and blood (CNR_scar-blood_ = 31.3 and 24.3 for blood and myocardium nulling respectively) as shown in Fig. 2d.

### Patients

**Fig. 3:**
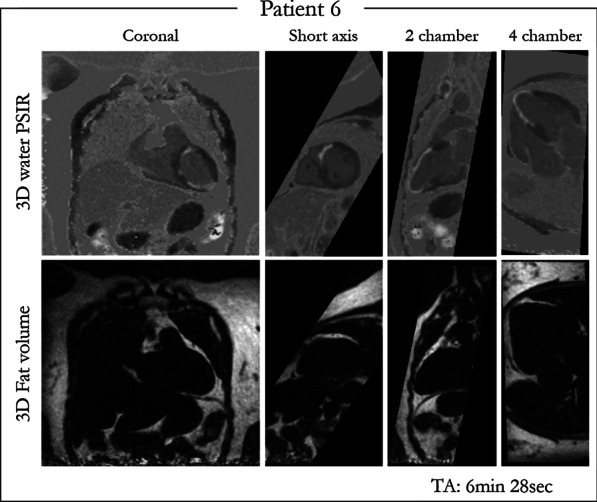
Co-registered 3D grey-blood PSIR LGE and fat volume obtained with the proposed approach and reformatted in different orientations (coronal, 2-chamber, 4-chamber and short-axis views) for one representative patient. Good depiction of scar is achieved in all the reformatted views. Good water/fat separation is obtained across the entire 3D volume. Contrast rations of CR_blood-myoc_ = 1.11, CR_scar-myoc_ = 1.32 and _CRscar-blood_ = 1.19 were measured

Intrinsically co-registered grey-blood 3D PSIR LGE and fat volumes reformatted in LV short-axis, 2-chamber and 4-chamber orientations are shown for one representative patient in Fig. 3. The patient showed a scar involving the LV mid-to-apical anterior wall, interventricular septum and true apex. Good scar depiction was observed in all the reformatted views with contrast ratio between scar, myocardium and blood of CR _blood-myoc_ = 1.11, CR_scar-myoc_ = 1.32 and CR_scar-blood_ = 1.19. Water/fat separation was achieved through the entire 3D volume with no evident swaps around the cardiac region. Fat signal in the water PSIR reconstructed image appears grey due to its nulling in the magnitude water IR-image.

**Fig. 4:**
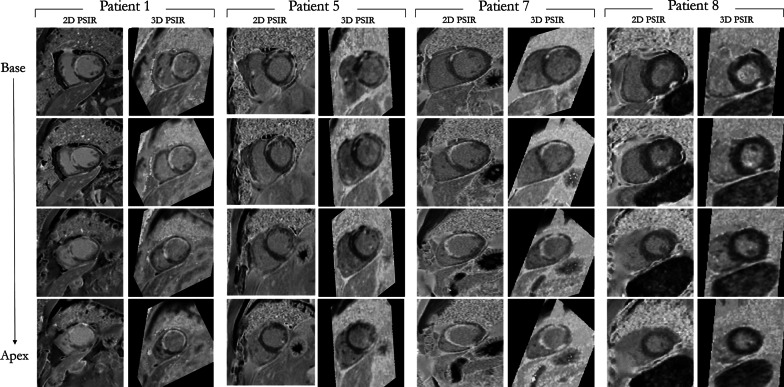
Qualitative comparison between grey-blood PSIR LGE images obtained with the proposed 3D sequence and 2D clinical acquisition for four patients. The acquired 3D volumes were reformatted to the same slice position as the 2D images acquired in short-axis. Good scar depiction is observed with the proposed technique in comparison to the 2D acquisition for all the patients

**Fig. 5:**
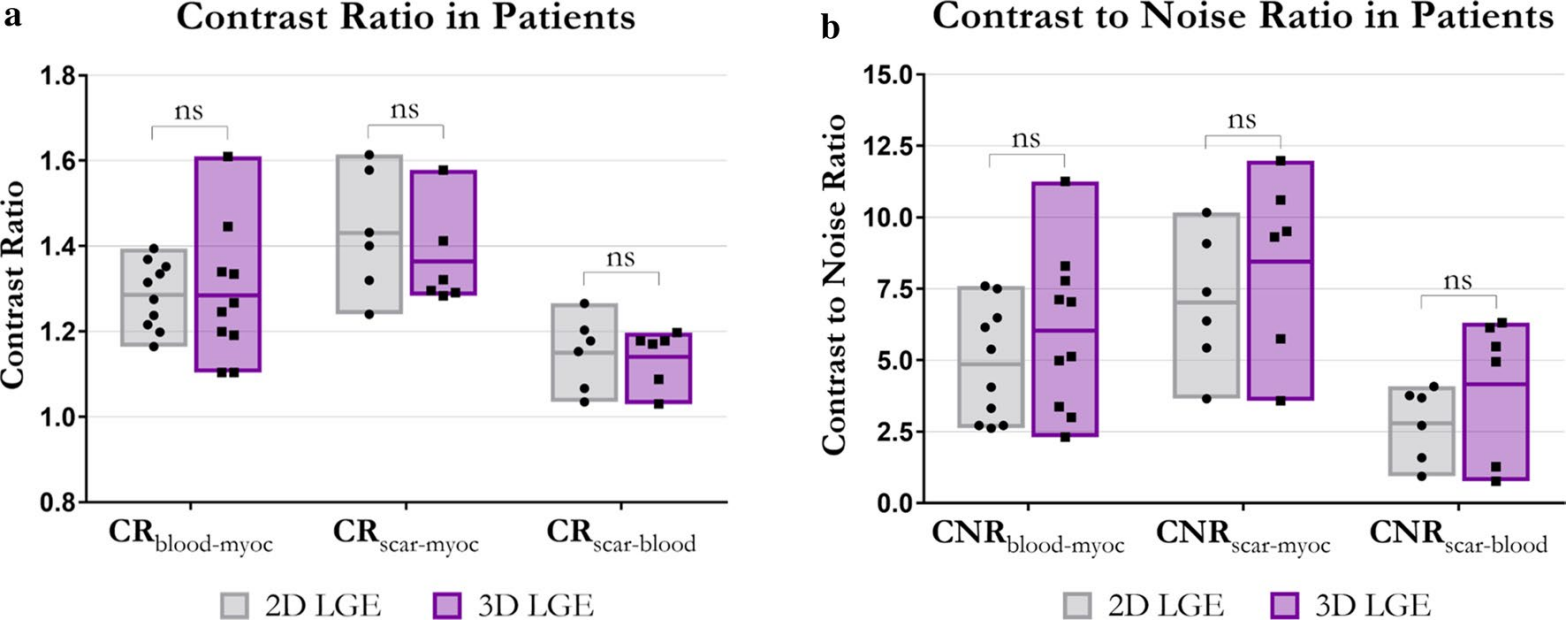
**a** Contrast ratio (CR) and **b** contrast-to-noise ratio (CNR) between blood-myocardium, scar-myocardium and scar-blood obtained with the 2D (grey) and 3D (purple) approaches. Blood-myocardium CR and CNR were measured on 10 acquired patients, whereas the scar-myocardium and scar-blood CR and CNR were measured on the 6 patients with scar. No significant differences were observed between the CRs and CNRs obtained with 2D and 3D approaches

The total acquisition times for 3D and 2D PSIR LGE were 6 min 48 s (range: 5 min 29 s*–*8 min 32 s) and 6 min 29 s (range: 6 min*–*7 min 10 s) respectively, with no statistical difference observed (p = 0.29). Comparison between short axis images obtained with the proposed grey-blood 3D PSIR LGE acquisition and the conventional 2D sequence are shown for four representative patients with scar in Fig. 4. Four short axis slices from the LV base to the apex are displayed for both 2D and 3D acquisitions; the 2D images were acquired in short axis orientation whereas the 3D volumes were reformatted to the corresponding short-axis images. Comparable image quality and scar depiction and delineation are found (Fig. 4) between the proposed 3D PSIR LGE sequence and the 2D PSIR LGE. CR and CNR between blood, myocardium and scar are shown in Fig. 5a, b respectively for the proposed 3D and 2D LGE acquisitions. The 2D and 3D LGE acquisitions showed similar CR between blood and myocardium (CR_2Dblood-myoc_ = 1.28 ± 0.08, CR_3Dblood-myoc_ = 1.28 ± 0.16; *p* = 0.98) and between scar and blood (CR_2Dscar-blood_ = 1.15 ± 0.08, CR_3Dscar-blood_ = 1.14 ± 0.06; *p* = 0.83). A trend towards a lower contrast ratio between scar and myocardium was observed with the 3D acquisition (CR_3Dscar-myoc_ = 1.36 ± 0.12) in comparison to the 2D sequence (CR_2Dmyoc-scar_ = 1.43 ± 0.14), although no statistical significant (*p* = 0.40). Higher CNRs between blood, myocardium and scar were obtained with the 3D LGE approach (CNR_3Dblood-myoc_ = 6.01 ± 2.80 CNR_3Dscar-myoc_ = 8.45 ± 3.20 and CNR_3Dscar-blood_ = 4.17 ± 4.10) in comparison to 2D grey-blood LGE acquisition (CR_2Dblood-myoc_ = 4.85 ± 2.00, CNR_2Dscar-myoc_ = 7.00 ± 2.40 and CNR_2Dscar-blood_ = 2.79 ± 1.30), although no statistical differences (*p* = 0.29, 0.40, 0.26 respectively for blood-myoc, scar-myoc and scar-blood) were observed between the two acquisitions. CR and CNR quantification for both 2D and 3D acquisitions are summarized in Table 2.

**Table 2:** Average contrast ratio (CR) and contrast-to-noise (CNR) ratio measured between blood-myocardium, scar-myocardium and scar-blood for both 2D and 3D LGE PSIR images

	2D		3D	
	CR	CNR	CR	CNR
Blood-myocardium	1.29 ± 0.08	4.85 ± 2.01	1.28 ± 0.16	6.03 ± 2.78
Scar-myocardium	1.43 ± 0.15	7.02 ± 2.4	1.36 ± 0.12	8.45 ± 3.16
Scar-blood	1.15 ± 0.09	2.79 ± 1.29	1.14 ± 0.07	4.15 ± 2.48

**Fig. 6:**
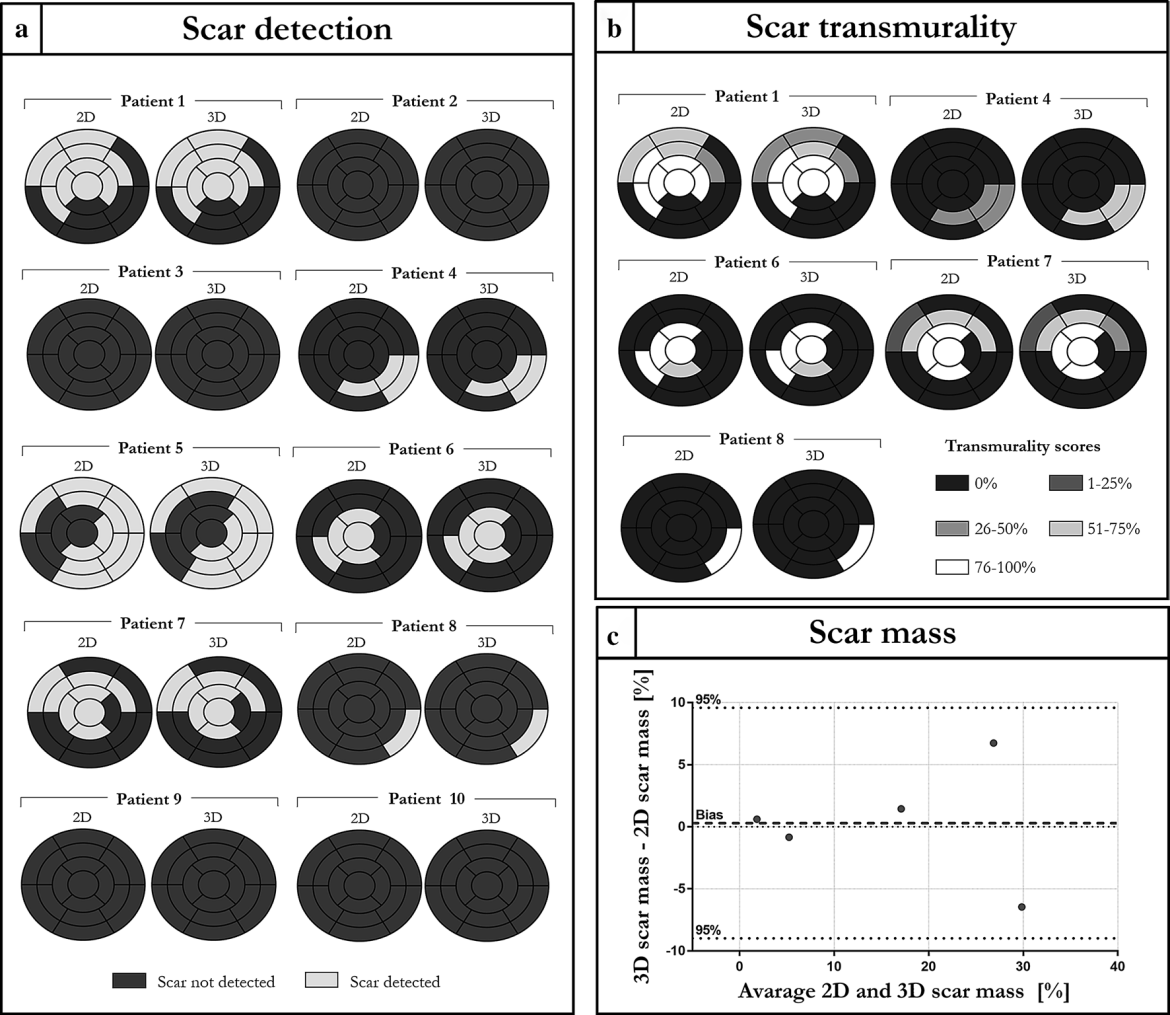
Analysis performed by observer 1. **a** Expert image analysis for scar detection with the 17 segment American Heart Association (AHA) model. **b** Scar transmuarlity score performed on the patients showing a myocardial scar. Patient 5 was excluded from the analysis because contrast retainment was due to non-ischemic cardiomyopathy. **c** Comparison between 2 and 3D measurement of scar mass performed via Bland Altman analysis

Scar detection, scar transmurality and scar mass assessment performed by the two observers are shown in Fig. 6 and Additional file 1: Figure S1 respectively.

Excellent agreement in scar detection and scar location between 2 and 3D acquisition with respect to the 17-segment American Heart Association (AHA) model were obtained (Fig. 6a and Additional file 1: Figure S1a). Good agreement in scar transmurality assessment (Fig. 6b and Additional file 1: Figure S1b) was obtained with the proposed 3D approach with respect to the 2D acquisition. K coefficients of 0.786 (*p* > 0.99) and 0.681 (*p* > 0.99) were obtained for the scar transmurality assessment for observer 1 and 2 respectively.

Good agreement in scar mass quantification, with no significant statistical difference (*p* = 0.89), was obtained for the 2D and 3D approaches. Biases of 0.3 and 1.2% were obtained for the measurements performed by the two observers respectively, with no data exceeding the 95% confidential intervals as shown in Fig. 6c and Additional file 1: Figure S1c.

Comparable image quality scores (Additional file 1: Figure S1) were obtained for the 2D and 3D LGE approaches. Mean image quality scores of 3.8 ± 0.4 and 3.6 ± 0.7 (*p* > 0.99) were obtained for 2D and 3D LGE acquisition respectively analysed by observer 1, whereas mean image quality scores of 4.0 ± 0.0 and 3.9 ± 0.3 (*p* > 0.99) were obtained for the 2D and 3D dataset analysed by observer 2. A comparison of per patient quality score is shown in Additional file 1: Figure S2. An inferior quality score was obtained with the proposed method only for Patient 3 due to water/fat swaps in subcutaneous fat regions, which however did not affect scar detection.

Intra-observer variability for scar detection was computed for each patient considering each LV segment in the analysis, and for all patients considering each segment of each patient in the analysis (Table 3). Intra-observer agreement was excellent for most of the patients in terms of scar detection and for all patients with regard to image quality assessment for both the 2D and 3D acquisition (Cohen’s kappa = 1). In patient 1 and 6 good agreement (0.6 < k < 0.8) was achieved whereas high agreement was observed for patient 8 (k > 0.8) for both the 2D and 3D datasets, leading to very good agreement in the overall patient scar detection k coefficient (k > 0.8).

**Table 3:** Intra-observer variability Cohen's kappa scores of scar detection and overall image quality score. The intra-observer variability analysis was performed for both 2D and 3D grey-blood LGE datasets

Intra-observer Variability					
		2D		3D	
	Patient no	Cohen kappa	*P*-value	Cohen kappa	*P*-value
Scar detection	1	0.658	0.95	0.658	0.95
	2	1	–	1	–
	3	1	–	1	–
	4	1	–	1	–
	5	1	–	1	–
	6	0.746	0.99	0.746	0.99
	7	0.883	0.99	0.883	0.99
	8	1	–	1	–
	9	1	–	1	–
	10	1	–	1	–
	All patients	0.898	0.99	0.898	0.99
Quality score	All patients	1	–	1	–

Intra-observer variability for scar detection was computed for each patient considering each left ventricular (LV) segment in the analysis, and for all patients considering each segment of each patient in the analysis

The results for inter-observer variability for scar detection, scar transmurality and image quality are shown in Table 4. Good to excellent agreement in terms of scar detection was achieved for each patient for both the 2D and 3D dataset, leading to excellent agreement obtained for the overall patient k-coefficient (k > 0.8).

**Table 4:** Inter-observer variability assessment for scar detection, scar transmurality and overall quality score

Inter-observer variability					
		2D		3D	
	Patient no	Cohen kappa	*P*-value	Cohen kappa	*P*-value
Scar detection	1	0.811	0.99	0.811	0.99
	2	1	–	1	–
	3	1	–	1	–
	4	1	–	1	–
	5	0.598	0.99	0.598	0.99
	6	0.638	0.99	0.638	0.99
	7	0.541	0.95	0.541	0.95
	8	0.638	0.95	0.638	0.95
	9	1	–	1	–
	10	1	–	1	–
	All patients	0.811	0.99	0.811	0.99
Scar transmurality	1	0.667	0.99	0.667	0.99
	4	0.622	–	0.805	–
	6	0.497	0.99	0.485	0.99
	7	0.512	0.99	0.439	0.99
	All patients with scar	0.589	0.99	0.544	0.99
Quality score	All patients	0.615	0.95	0.5	0.95

The inter-observer variability analysis was performed for both 2D and 3D grey-blood late gadolinium enhancement (LGE) datasets. Inter-observer variability for scar detection was computed for each patient considering each LV segment in the analysis, ad for all patients considering each segment of each patient in the analysis. Inter-observer variability of scar transmurality was performed only for patients showing a myocardial scar

Good agreement in scar transmurality was obtained for patient 1 and 4 for both 2D and 3D dataset, whereas a fair agreement was obtained for patients 6 and 7, leading to an overall fair agreement in terms of scar transmurality between the two observers for both 2D and 3D datasets. Good and fair agreement in terms of image quality score were obtained respectively for 2D (k = 0.615) and 3D (k = 0.5) acquisitions. Inter-observer variability of scar mass quantification was performed with Bland Altman analysis as shown in Additional file 1: Figure S3 for 2D and 3D acquisitions. Biases of -1.6% and -0.7% were observed for the 2D and 3D approaches respectively.

The need for using 2D iNAVs to independently correct for respiratory motion in both the IR-prepared and reference heartbeats is shown in Additional file 1: Figure S4 for one representative patient. PSIR image calculated from motion corrected IR and reference images resulted in a better scar delineation in comparison to PSIR reconstruction with motion correction applied to the IR image only.

## Discussion

In this work we present a free-breathing motion corrected sequence for the acquisition of isotropic-resolution grey-blood 3D PSIR LGE and complementary 3D fat volumes in a total scan time of < 7 min.

The proposed approach extends a previously introduced grey-blood 2D PSIR LGE sequence that has shown to increase the CR between scar and blood by nulling the blood in the IR-magnitude image, leading to better scar depiction in the presence of subendocardial scarring [20]. Extending the 2D framework to a 3D acquisition required some key components. A 3D variable density spiral like Cartesian trajectory with acceleration factor of 3 × was used for undersampled data acquisition. iNAVs were used to track and correct SI and LR respiratory motion for both IR-prepared and reference images enabling 100% respiratory scan efficiency and predictable scan time, as well as motion corrected PSIR reconstruction thereby minimising misregistration between the two datasets. The combination of undersampling and motion compensation enabled the acquisition of grey-blood 3D PSIR and fat volumes in clinically affordable scan time of < 7 min minimising contrast wash-out that could affect image quality for acquisitions longer than 10 min. Minimising additional contrast agent wash-out during image acquisition was particularly relevant in this study due to the suboptimal acquisition starting time of the 3D approach (~ 25 min after gadolinium injection). The proposed 3D sequence was acquired at the end of the clinical protocol to avoid interference with the acquisition of clinically relevant data. Despite the suboptimal post contrast imaging time point, contrast between scar and blood and scar and myocardium was comparable to the 2D grey-blood PSIR sequence. PSIR LGE imaging has been shown to overcome limitations of inaccurate TI selection related to magnitude LGE reconstruction which may have been a limitation of recently published 3D LGE methods [24, 25]. Finally, the acquisition was performed with a dual-echo Dixon encoded GRE readout. Water/fat separation permitted to increase the signal of the water images [37] and to enable distinct visualization of LGE and fat signals.

The proposed grey-blood 3D PSIR LGE (magnitude-blood nulling) approach was compared to conventional magnitude myocardium-nulling (bright-blood PSIR) in a phantom experiment in terms of CR and CNR between blood, scar and myocardium. A higher scar-to-blood contrast was observed in the PSIR images obtained with the proposed approach while scar-to-myocardium contrast was maintained. These results were consistent with those observed by Holtackers et al. [20], thus in-vivo comparison was carried out between the 2D grey-blood PSIR approach and the proposed 3D extension.

Ten patients (6 with myocardialscar) were acquired with the proposed grey-blood 3D PSIR LGE sequence and conventional grey-blood 2D PSIR LGE approach with no statistically significant difference in total acquisition time between both methods. The 3D isotropic acquisition enabled whole-heart coverage, which is beneficial for the quantification of the scar identification and quantification [24, 25], and permitted the reformatting of the PSIR and fat volumes in different orientations maintaining good image quality. This allows to circumvent the drawbacks related to 2D acquisition such as limited coverage, misalignment between different slices and grossly anisotropic resolution. Additionally the complementary fat volume obtained from Dixon water/fat separation could facilitate the assessment of fibro-fatty infiltration in the myocardium alongside a higher confidence in discriminating between subepicardial LGE and epicardial fat [27, 28].

The proposed approach showed comparable CR and CNR between scar, blood and myocardium with respect to conventional grey-blood 2D sequence. A tendency towards higher CNR was observed with the proposed 3D as compared to 2D approach likely due to increased SNR obtained with the Dixon encoded acquisition. Excellent inter-observer agreement in scar detection, good agreement in scar transmurality and scar mass and comparable image quality scores were obtained both for 2D and 3D sequences. Excellent intra-observer agreement was observed both for scar detection and image quality.

An overall decrease in scar sharpness was visually observed with the proposed 3D sequence, likewise due to lower in-plane resolution for 3D (2 mm) in comparison to the 2D (1.4 mm) acquisition. Besides, residual respiratory motion could also affect scar depiction and sharpness. Of importance, the use of iNAVs enabled efficient correction of translational respiratory motion for both the IR-prepared and reference datasets independently (Additional file 1: Figure S4) leading to clinically affordable scanning time.

## Limitations

Although good motion correction was achieved with the proposed method, a potential limitation of this work is the assumption that respiratory motion is purely translational in SI and LR direction. Anterior–posterior, rotational and non-rigid motion of the heart could generate residual motion in the IR-prepared and reference dataset and thus could affect the quality of the reconstructed PSIR images. Non-rigid motion correction techniques should be investigated and incorporated in the reconstruction framework in future work [41, 42] to minimise residual non-rigid motion. Additionally, a free breathing CINE scan should be performed immediately before the 3D grey-blood LGE acquisition to account for any variability in the patient heart rate. Furthermore, the impact of higher spatial resolution grey-blood 3D PSIR LGE readouts should be investigated in further dedicated studies. Finally, the acquisition of the 3D dataset was performed at the end of the clinical protocol, thus a suboptimal contrast between scar and healthy myocardium may have been observed due to GBCA wash out. Although, no statistical difference was observed in CR_scar-myoc_ and CNR_scar-blood_ measurement in patients, 2D and 3D PSIR LGE sequence should be acquired in randomized order in future studies to investigate the influence of the time between GBCA administration and sequence acquisition on image quality, CNR and scar detection.

## Conclusion

We present a free-breathing 3D sequence with magnitude blood nulling that enabled the acquisition of isotropic resolution grey-blood PSIR LGE images and a co-registered fat volume in a total scan time of < 7 min. The proposed sequence showed high contrast between scar, blood and myocardium and excellent agreement in scar depiction in comparison to conventional grey-blood 2D PSIR LGE. Future work will investigate the acquisition of datasets with increased isotropic resolution in a larger cohort of patients and incorporation of non-rigid motion correction to further improve scar depiction.

## Supplementary Information

**Additional file 1: Figure S1.** Analysis performed by observer 2. **a** Expert image analysis for scar detection with the 17 segment AHA model. **b** Scar transmuarlity score performed on the patients showing a myocardial scar. Patient 5 was excluded from the analysis because contrast retainment was due to non-ischemic cardiomyopathy. **c** Comparison between 2 and 3D measurement of scar mass performed via Bland Altman analysis. **Figure S2.** Expert image quality assessment (1: non-diagnostic, 2: poor, 3: good and 4: excellent diagnostic quality) for all the acquired patients. Comparable results were obtained with the 3D and 2D approaches by both observers. An inferior quality score was obtained with the proposed method only for Patient 3 due to water/fat swaps in subcutaneous fat regions, which however did not affect scar detection. **Figure S3**. Inter-observer variability of scar mass quantification for 2D and 3D LGE PSIR acquisitions. Inter-observer variability was quantified via Bland Altman analysis of scar mass measurements performed by the two observers for both the 2D and 3D grey-blood LGE PSIR acquisitions. **Figure S4.** Effect of motion correction of IR and reference datasets on the PSIR reconstructed images. Top row: PSIR image calculated from motion corrected IR and reference volumes. Bottom row: PSIR image calculated from motion corrected IR volume and no motion corrected reference volume. Sharp scar delineation is obtained in the PSIR image obtained with motion correction performed on both IR-prepared and reference volume (blue line). Impaired scar delineation is obtained in the PSIR image reconstructed performing the motion correction only on the IR-prepared dataset as shown from the signal intensity profile across the left ventricle (orange line).

## Data Availability

The datasets generated and/or analysed during the current study are not publicly available due information content that could compromise research participant privacy/consent, but are available from the corresponding author on reasonable request.

## Abbreviations

AHA: American Heart Association
bSSFP: Balanced steady state free precession
CMR: Cardiovascular magnetic resonance
CNR: Contrast-to-noise ratio
CR: Contrast ratio
ECG: Electrocardiogram
GBCA: Gadolinium based contrast agent
GRE: Gradient echo
iNAV: Image navigator
IR: Inversion recovery
IRSE: Inversion recovery spin echo
LGE: Late gadolinium enhancement
LR: Left–right
LV: Left ventricle/left ventricular
PSIR: Phase sensitive inversion recovery
SI: Superior-inferior
SNR: Signal-to-noise ratio
TI: Inversion time
T2prep: T2 preparation
