# Supplementary material for: 3D whole-heart grey-blood late gadolinium enhancement cardiovascular magnetic resonance imaging
Source: J Cardiovasc Magn Reson. 2021 May 24;23:62. doi: 10.1186/s12968-021-00751-2 (PMC8142497; doi:10.1186/s12968-021-00751-2)
Supplement: Supplementary file 1 — Additional file 1: Figure S1. Analysis performed by observer 2. a Expert image analysis for scar detection with the 17 segment AHA model. b Scar transmuarlity score performed on the patients showing a myocardial scar. Patient 5 was excluded from the analysis because contrast retainment was due to non-ischemic cardiomyopathy. c Comparison between 2 and 3D measurement of scar mass performed via Bland Altman analysis. Figure S2. Expert image quality assessment (1: non-diagnostic, 2: poor, 3: good and 4: excellent diagnostic quality) for all the acquired patients. Comparable results were obtained with the 3D and 2D approaches by both observers. An inferior quality score was obtained with the proposed method only for Patient 3 due to water/fat swaps in subcutaneous fat regions, which however did not affect scar detection. Figure S3. Inter-observer variability of scar mass quantification for 2D and 3D LGE PSIR acquisitions. Inter-observer variability was quantified via Bland Altman analysis of scar mass measurements performed by the two observers for both the 2D and 3D grey-blood LGE PSIR acquisitions. Figure S4. Effect of motion correction of IR and reference datasets on the PSIR reconstructed images. Top row: PSIR image calculated from motion corrected IR and reference volumes. Bottom row: PSIR image calculated from motion corrected IR volume and no motion corrected reference volume. Sharp scar delineation is obtained in the PSIR image obtained with motion correction performed on both IR-prepared and reference volume (blue line). Impaired scar delineation is obtained in the PSIR image reconstructed performing the motion correction only on the IR-prepared dataset as shown from the signal intensity profile across the left ventricle (orange line). [file 12968_2021_751_MOESM1_ESM.docx]

**Supporting Information Figures**


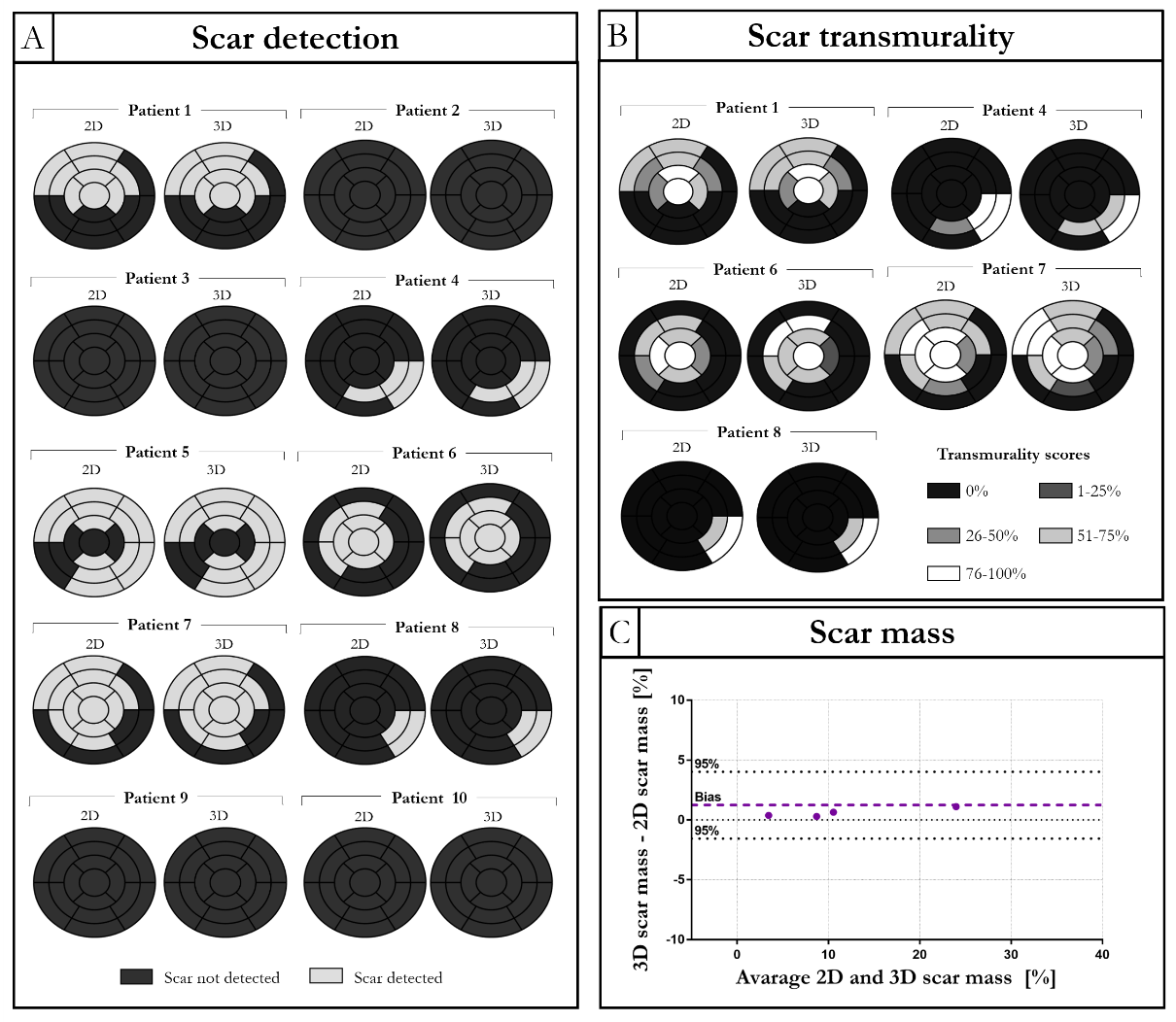


*Supporting Information Figure S1 – Analysis performed by observer 2. A) Expert image analysis for scar detection with the 17 segment AHA model. B) Scar transmuarlity score performed on the patients showing a myocardial scar. Patient 5 was excluded from the analysis because contrast retainment was due to non-ischemic cardiomyopathy. C) Comparison between 2D and 3D measurement of scar mass performed via Bland Altman analysis.*


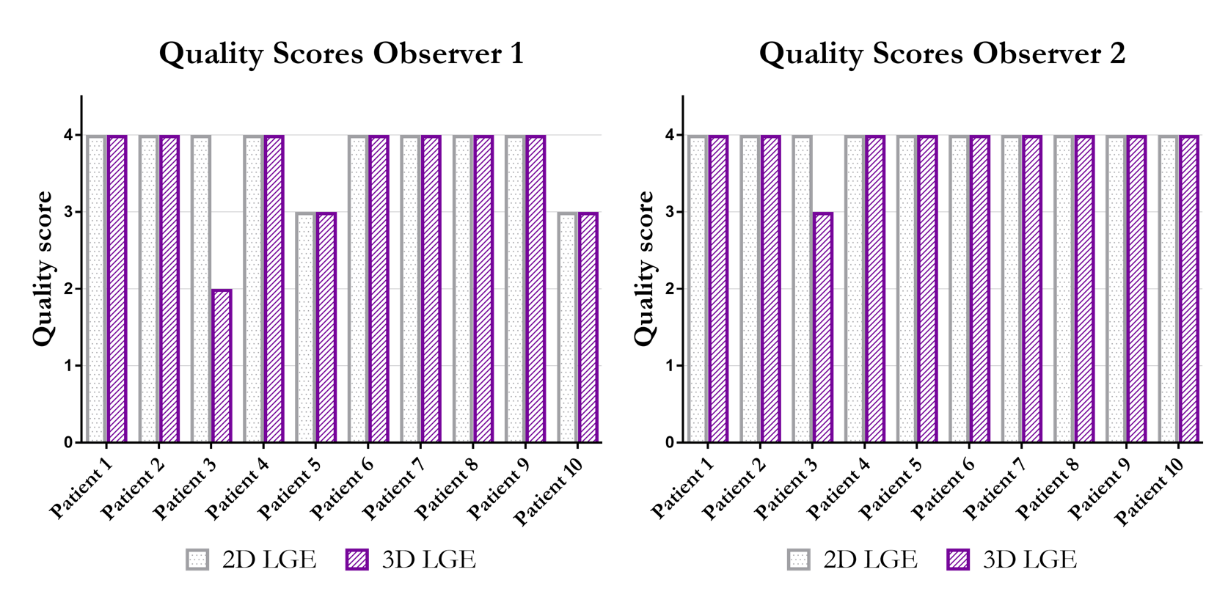


*Supporting Information Figure S2 – Expert image quality assessment (1: non-diagnostic, 2: poor, 3: good and 4: excellent diagnostic quality) for all the acquired patients. Comparable results were obtained with the 3D and 2D approaches by both observers. An inferior quality score was obtained with the proposed method only for Patient 3 due to water/fat swaps in subcutaneous fat regions, which however did not affect scar detection.*


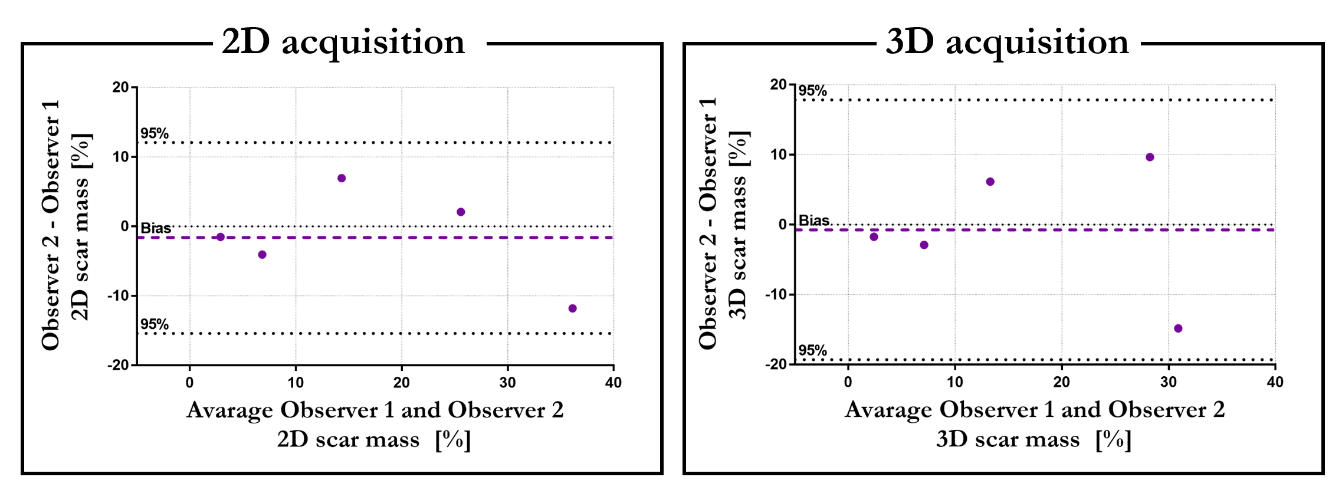


*Supporting Information Figure S3 – Inter-observer variability of scar mass quantification for 2D and 3D LGE PSIR acquisitions. Inter-observer variability was quantified via Bland Altman analysis of scar mass measurements performed by the two observers for both the 2D and 3D grey-blood LGE PSIR acquisitions.*


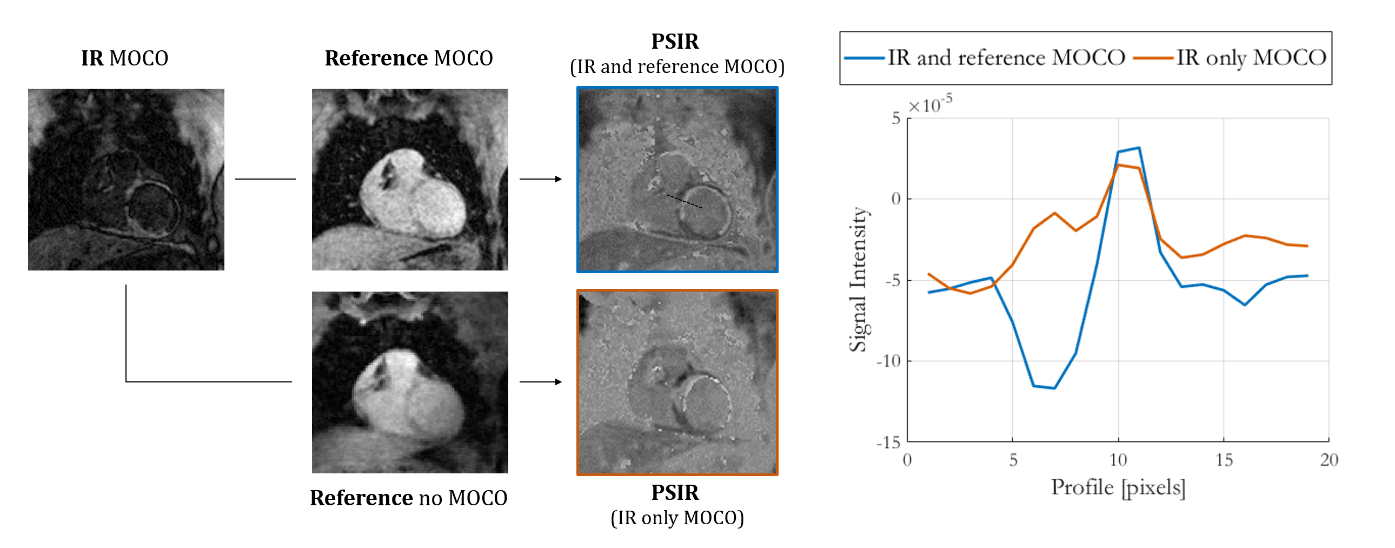


Supporting Information Figure S4 – Effect of motion correction of IR and reference datasets on the PSIR reconstructed images. Top row: PSIR image calculated from motion corrected IR and reference volumes. Bottom row: PSIR image calculated from motion corrected IR volume and no motion corrected reference volume. Sharp scar delineation is obtained in the PSIR image obtained with motion correction performed on both IR-prepared and reference volume (blue line). Impaired scar delineation is obtained in the PSIR image reconstructed performing the motion correction only on the IR-prepared dataset as shown from the signal intensity profile across the left ventricle (orange line).
